# Verschiedene immunologische Typen der CRSwNP im Kontext der neuen Europäischen EAACI-Nomenklatur: Teil 3: Hypersensitivitätsreaktionen vom Typ VI

**DOI:** 10.1007/s00106-026-01732-6

**Published:** 2026-02-03

**Authors:** L. Klimek, S. Becker, B. Haxel, M. Cuevas, P. Huber, A. Chaker, O. Pfaar, M. Laudien, C. Beutner, J. Hagemann, U. Förster-Ruhrmann, H. Olze, B. P. Ernst, A. Beule, C. Rudack, A. S. Hoffmann, C. Betz, M. Gröger

**Affiliations:** 1 Zentrum für Rhinologie und Allergologie, Wiesbaden, Deutschland; 2 Klinik für Hals‑, Nasen- und Ohrenheilkunde, Universitätsmedizin Tübingen, Tübingen, Deutschland; 3 Klinik für Hals‑, Nasen- und Ohrenheilkunde, Universitätsmedizin Mainz, Mainz, Deutschland; 4 Klinik und Poliklinik für HNO-Heilkunde, Medizinische Fakultät und Universitätsklinikum Carl Gustav Carus, TU Dresden, Dresden, Deutschland; 5 Klinik und Poliklinik für Hals-Nasen- Ohrenheilkunde, Klinikum der Ludwig-Maximilians-Universität München, München, Deutschland; 6 TUM School of Medicine and Health, Klinikum rechts der Isar, HNO-Klinik und Zentrum für Allergie und Umwelt, Technische Universität München, München, Deutschland; 7 Klinik für Hals‑, Nasen- und Ohrenheilkunde, Universitätsklinikum Gießen und Marburg GmbH, Standort Marburg, Marburg, Deutschland; 8 Klinik für Hals‑, Nasen- und Ohrenheilkunde, Universitätsklinikum Kiel, Kiel, Deutschland; 9 Klinik für Dermatologie, Venerologie und Allergologie, Universitätsmedizin Göttingen, Göttingen, Deutschland; 10 Charité – Universitätsmedizin Berlin, Berlin, Deutschland; 11 Klinik für Hals‑, Nasen- und Ohrenheilkunde, Universitätsmedizin Frankfurt/M., Frankfurt/M., Deutschland; 12 Klinik für Hals‑, Nasen- und Ohrenheilkunde, Universitätsklinikum Münster, Münster, Deutschland; 13 Klinik für Hals‑, Nasen- und Ohrenheilkunde, Universitätsklinikum Hamburg-Eppendorf, Hamburg, Deutschland

**Keywords:** Immunologische Endotypen, T2-Endotyp, Chemisch induzierte Entzündung, IL-33/TSLP (Alarmine), PGE2-Synthese, Immunological endotypes, T2 endotype, Chemically induced inflammation, IL-33/TSLP (alarmins), PGE2 synthesis

## Abstract

**Hintergrund:**

Die chronische Rhinosinusitis (CRS) weist in Europa und den USA eine Prävalenz von bis zu 11 % auf und gehört somit zu den häufigsten chronischen Erkrankungen überhaupt. Die Klassifizierung nach immunologischen Endotypen findet immer mehr Eingang in die Krankheitsdefinition, vor allem bei der chronischen Rhinosinusitis mit Nasenpolypen (CRSwNP). Je nach den spezifischen Mechanismen für die chronische Gewebeentzündung werden verschiedene Endotypen charakterisiert. Hierbei spielen genetische und epigenetische Veränderungen im mukosalen Immunsystem eine wichtige Rolle. Die Identifizierung von Endotypen kann dazu beitragen, die Heterogenität innerhalb der Erkrankung zu verstehen und personalisierte Behandlungsansätze zu entwickeln. Im Teil 1 dieser Publikation hatten wir die immunologischen Klassifizierungen der Hypersensitivitätsreaktionen vom Typ IV (T1-, T2-, und T3-Endotypen), in Teil 2 die Hypersensitivitätsreaktionen vom Typ V (epitheliale Barriereschädigungen) dargestellt.

Ziel des nun vorliegenden Teils 3 dieser Übersichtsarbeit ist es, die Hypersensitivitäts-Immunreaktionen vom Typ VI darzustellen und ihre Implikationen für eine erweiterte Diagnostik und Therapie aufzuzeigen.

**Methodik:**

Die Europäische Akademie für Allergie und klinische Immunologie (EAACI) hat in einem kürzlich erschienenen Positionspapier eine Aktualisierung der Nomenklatur für immunologische Überempfindlichkeitsreaktionen auf der Basis von neun verschiedenen immunologischen Reaktionstypen vorgestellt. Die ursprünglich von Coombs und Gell klassifizierten Antikörper-vermittelten Reaktionen vom Typ I, Typ II und Typ III wurden erweitert und detailliert beschrieben. Direkte zelluläre und entzündliche Reaktionen auf chemische Substanzen werden als Typ VI dieser Hypersensitivitätsreaktionen definiert und nun in Teil 3 dieser Publikationsreihe erörtert.

**Ergebnisse:**

Bei der CRSwNP mit Hypersensitivitäts-Immunreaktionen vom Typ VI findet sich ein Ungleichgewicht zwischen den Cyclooxygenase-Isoformen 1 und 2, das sich durch COX-1-hemmende Medikamente verschlechtert und zu einer verminderten PGE2-Synthese und einer Überproduktion von LTC4 und PGD2 führt. Nachfolgend werden im Rahmen der chronischen Schleimhautentzündung Alarmine wie Interleukin-33 (IL-33) und thymisches stromales Lymphopoietin (TSLP) freigesetzt. Diese Zytokine aktivieren Th2-Lymphozyten und Innate Lymphoid Cells Typ 2 (ILC2) zur Freisetzung von Zytokinen wie IL‑4, IL‑5 und IL-13.

**Schlussfolgerung:**

Die Erweiterung der immunologischen Klassifikation auf Hypersensitivitätsreaktionen vom Typ VI liefert einen wichtigen Beitrag zum besseren Verständnis der Pathophysiologie der chronischen Rhinosinusitis mit Nasenpolypen (CRSwNP). Die Identifikation dieser spezifischen Reaktionsmuster – insbesondere der durch chemische Substanzen induzierten Entzündungsprozesse – verdeutlicht die Komplexität der zugrunde liegenden Immunmechanismen und unterstreicht die Notwendigkeit einer endotypenbasierten Diagnostik. Die Einbindung dieser Erkenntnisse in die klinische Praxis ermöglicht eine gezieltere, individualisierte Therapie und stellt einen weiteren Schritt in Richtung personalisierter Medizin bei CRS dar.

## Abstract (en)

**Background:**

Chronic rhinosinusitis (CRS) has a prevalence of up to 11% in Europe and the USA, making it one of the most common chronic diseases. Classification based on immunological endotypes is increasingly being integrated into the disease definition, particularly for chronic rhinosinusitis with nasal polyps (CRSwNP). Depending on the specific mechanisms underlying chronic tissue inflammation, different endotypes are characterized. Genetic and epigenetic changes in the mucosal immune system play a significant role in this context. Identifying endotypes can help to better understand disease heterogeneity and develop personalized treatment approaches. In part 1 of this publication, we discussed the immunological classifications of type IV hypersensitivity reactions (T1-, T2-, and T3-endotypes), while part 2 focused on type V hypersensitivity reactions (epithelial barrier defects). The aim of part 3 is to describe type VI hypersensitivity immune reactions and highlight their implications for extended diagnostics and treatment.

**Methods:**

The European Academy of Allergy and Clinical Immunology (EAACI) recently published a position paper presenting an updated nomenclature for immunological hypersensitivity reactions, now encompassing nine distinct immunological reaction types. The antibody-mediated reactions originally classified by Coombs and Gell as types I, II, and III have been expanded and described in greater detail. Direct cellular and inflammatory responses to chemical substances are defined as type VI hypersensitivity reactions and are the focus of this third part of the publication series.

**Results:**

In CRSwNP patients with type VI hypersensitivity immune reactions, an imbalance between cyclooxygenase (COX) isoforms 1 and 2 can be observed. This imbalance is exacerbated by COX-1-inhibiting drugs, leading to reduced prostaglandin E2 (PGE2) synthesis and overproduction of leukotriene C4 (LTC4) and PGD2. As a result of the chronic mucosal inflammation, alarmins such as interleukin-33 (IL-33) and thymic stromal lymphopoietin (TSLP) are released. These cytokines activate Th2 lymphocytes and type 2 innate lymphoid cells (ILC2s), prompting the release of cytokines such as IL‑4, IL‑5, and IL-13.

**Conclusion:**

Expansion of the immunological classification to include type VI hypersensitivity reactions represents an important step toward a better understanding of the pathophysiology of CRSwNP. Identifying these specific reaction patterns—particularly those triggered by chemical substances—highlights the complexity of the underlying immune mechanisms and emphasizes the need for endotype-based diagnostics. Incorporating these insights into clinical practice allows for more targeted individualized therapies and marks another step toward personalized medicine in CRS.

Die chronische Rhinosinusitis (CRS) weist in Europa und den USA eine Prävalenz von bis zu 11 % auf und gehört somit zu den häufigsten chronischen Erkrankungen überhaupt [1]. Die Diagnose CRS beruht üblicherweise auf klinischen Parametern, wobei mindestens zwei der Hauptsymptome (Gesichts‑/Kopfdruck, nasale Obstruktion, Hyposmie/Anosmie und/oder Nasensekretion) oder ein Hauptsymptom und mindestens 2 Nebensymptome (Kopfschmerz, Fieber, Halitosis, Husten, Zahnschmerzen, Abgeschlagenheit und Ohrdruck) vorliegen sollten über einen Zeitraum von mehr als 12 Wochen. Des Weiteren wird ein endoskopischer und/oder radiologischer Nachweis entzündlichen Gewebes zusätzlich zu o. g. Kriterien gefordert. Die derzeit noch aktuelle deutsche S2k-Leitlinie Rhinosinusitis (AWMF 017/049 (HNO) und AWMF 053-012 (DEGAM)) unterscheidet zwischen einer akuten, einer rezidivierenden akuten und einer chronischen Rhinosinusitis (RS).

Unterschieden wird zwischen einer CRS mit (CRSwNP) und ohne Polypen (CRSsNP). Die CRSwNP ist mit einer Prävalenz von bis zu 4 % deutlich seltener als die CRSsNP, weist jedoch meist einen schwereren Krankheitsverlauf auf [1]. CRSwNP und CRSsNP sind für sich gesehen jeweils keine einheitlichen Krankheitsbilder, da innerhalb dieser Phänotypen verschiedene Pathomechanismen existieren, die zu unterschiedlichen inflammatorischen Reaktionen der Mukosa führen, welche als Endotypen bezeichnet werden [84]. Die CRS-Endotypen 1 bis 3 gemäß EPOS 2020 unterscheiden sich vor allem durch ihre dominierenden Immunreaktionen: T1-Endotypen sind geprägt durch eine Interferon-γ-dominierte Entzündung mit Aktivierung von Th1-Zellen, typisch für Abwehrreaktionen gegen intrazelluläre Erreger und häufig verbunden mit neutrophiler Inflammation. T2-Endotypenß zeigen eine eosinophile, von IL‑4, IL‑5 und IL-13 getragene Immunantwort mit Aktivierung von Th2-Zellen und ILC2 – dieser Endotyp ist besonders charakteristisch für CRSwNP, häufig assoziiert mit Asthma und systemischer Typ-2-Inflammation und therapeutisch relevant für den Einsatz von Biologika. T3-Endotypen wiederum sind durch eine IL-17- und IL-22-dominierte Immunaktivierung gekennzeichnet, die typischerweise mit neutrophiler Entzündung und Reaktionen auf bakterielle bzw. fungale Stimuli verknüpft ist [25].

Bei der CRSwNP spielen Inhalationsallergien eher eine untergeordnete Rolle [20, 30, 46, 116], wohingegen bei der CRSsNP Allergien, vor allem auf perenniale Antigene wie z. B. Milbenantigene, eine ursächliche Rolle spielen können und entsprechende Therapieoptionen bedacht und angewendet werden sollten [7, 8, 13, 14, 22, 43, 44, 61, 65, 93, 111, 113].

Die Bezeichnung „Überempfindlichkeit“ wurde erstmals 1963 von Robin Coombs und Philip George Houthem Gell eingeführt. Überempfindlichkeit bezieht sich auf eine unerwünschte, unangenehme oder schädliche Reaktion, die aus einer Überreaktion der adaptiven Immunantwort resultiert. Sie umfasst sowohl Allergien, die durch äußere Reize ausgelöst werden, als auch Autoimmunität, die auf intrinsische Reize zurückzuführen ist. Typischerweise setzen Überempfindlichkeitsreaktionen einen vorsensibilisierten (Immun‑)Zustand des Organismus voraus (sekundäre Immunantwort). Nach Coombs und Gell wurden Überempfindlichkeitsreaktionen in vier Typen eingeteilt: Typ I: unmittelbar (IgE-vermittelt), Typ II: zytotoxisch (antikörper- und Fc-Rezeptor-vermittelt, zellulär), Typ III: immunkomplexvermittelt, und Typ IV: verzögert (T-Zell-vermittelt) [21, 38–41]. Typ-V-Überempfindlichkeitsreaktionen werden bei Defekten der Epithelbarriere und mikrobieller Dysbiose gefunden. Zusätzlich hierzu führten neue Erkenntnisse zur Definition einer Hypersensitivitätsreaktion durch direkte zelluläre und inflammatorische Reaktionen auf chemische Substanzen (Typ VI), die auch bei der CRS existiert und Gegenstand der vorliegenden Übersichtsarbeit ist.

Das Verständnis der hier dargelegten pathophysiologischen Grundlagen soll in der Zukunft eine präzise, individualisierte Therapie ermöglichen.

## Methodik

Der vorliegenden Publikation liegt ein Positionspapier der Europäischen Akademie für Allergie und klinische Immunologie (EAACI) zugrunde, in dem eine modernisierte und erweiterte Klassifikation von Hypersensitivitätsreaktionen vorgestellt wird [39]. Diese wurde inzwischen auch an das deutsche Gesundheitssystem angepasst [38, 40, 41].

Die bei der CRSwNP bestehenden fehlgeleiteten immunologischen Hyperreaktivitätsreaktionen in der Mukosa der Nasennebenhöhlen werden in dieser Beitragsreihe vor dem Hintergrund der genannten neuen Klassifikation näher beleuchtet [1, 73, 74, 92]. Der vorliegende Beitrag widmet sich hierbei der Hypersensitivitätsreaktionen vom Typ VI.

## Ergebnisse

Die Aspirin-exazerbierte Atemwegserkrankung (AERD/NERD) ist eine chronisch-entzündliche Erkrankung, die im Fall einer typischen Ausprägung gekennzeichnet ist als ein Syndrom, bestehend aus einer Trias aus Asthma, wiederkehrenden Nasenpolypen und einer Überempfindlichkeit gegen Aspirin und andere NSAID mit Reaktionen der unteren und oberen Atemwege nach oraler, parenteraler oder sogar topischer Einnahme nichtsteroidaler entzündungshemmender Medikamente (NSAID). Die Krankheit wird derzeit international als NSAID-exazerbierte Atemwegserkrankung (NERD; Synonym: „aspirin-exacerbated respiratory disease“, AERD) bezeichnet. Früher wurden vor allem die folgenden Bezeichnungen für diese Krankheit verwendet: Widal-Syndrom, Samter-Syndrom, Samter-Trias, ASA-Trias oder Aspirinintoleranz [23, 50, 117]. Der zugrunde liegende Mechanismus wird unten näher beschrieben, wesentlich ist hierbei eine nichtallergische Überempfindlichkeit gegen alle die Cyclooxygenase (COX)-1 hemmenden NSAID [50].

Die Prävalenz der NERD in der Allgemeinbevölkerung liegt Berichten zufolge zwischen 0,5 und 5,7 % [105]. Frauen sind häufiger betroffen (60–70 %) als Männer, und die Krankheit könnte eine noch nicht endgültig aufgeklärte genetische Veranlagung haben [36, 89]. Darüber hinaus scheint die Dunkelziffer der NERD hoch zu sein: In einer Studie des European Network of Aspirin-induced Asthma [105] mit 500 Asthmapatienten wurde durch Provokationstests eine bisher unbekannte Analgetika-Intoleranz von 15 % festgestellt. Bei Vorliegen einer chronischen Rhinosinusitis wurde bei 34 % der Patienten eine NERD diagnostiziert [105]. Dieser Zusammenhang zwischen CRSwNP und Asthma ist insgesamt sehr häufig. Eine NERD betrifft etwa 12–40 % der Fälle von schwerem Asthma [3, 82] und bis zu 39 % der Fälle von schwerer CRSwNP [25, 97]. Darüber hinaus gibt es eine separate kutane Form der Analgetika-Intoleranz bei Patienten mit chronischer Urtikaria, die auch als „NSAID-exacerbated cutaneous disease“ (NECD) oder, im Fall von Urtikaria/Angioödem bei Patienten ohne kutane chronische Erkrankung, als „NSAID-induced urticaria/angioedema“ (NIUA) [52, 89] bezeichnet wird.

## Pathophysiologie der NERD

**Abb. 1:**
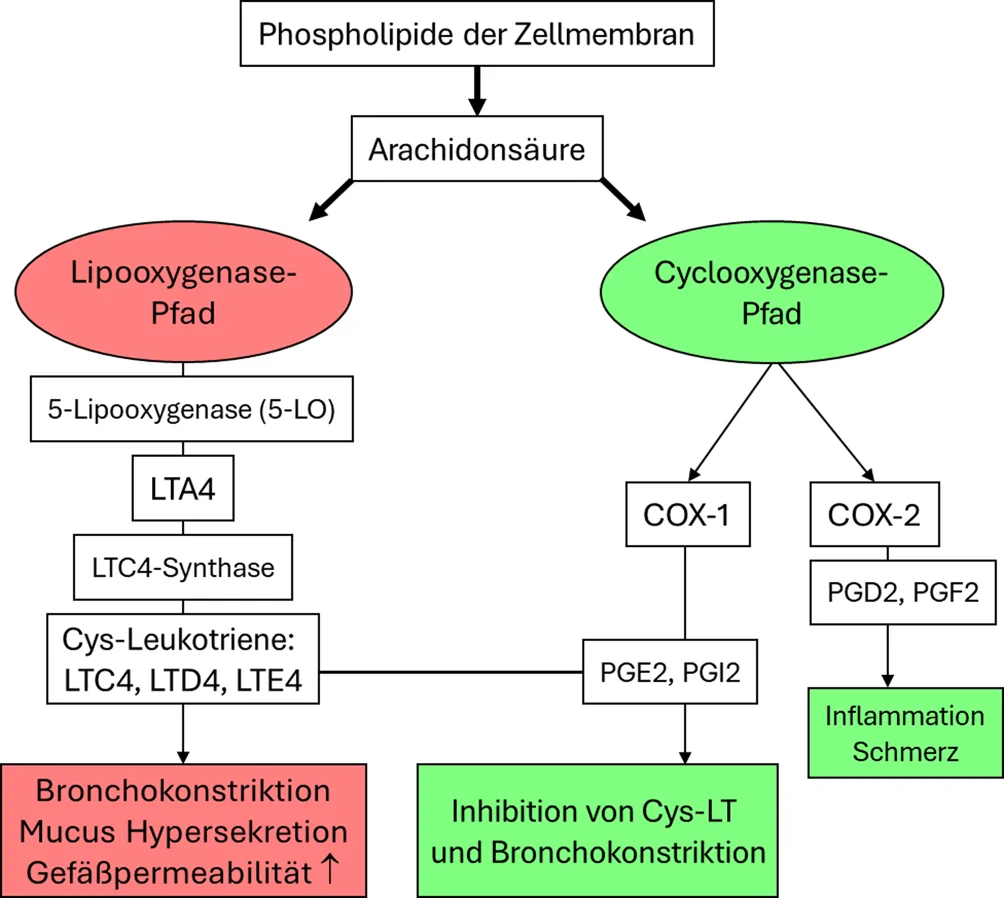
Pathophysiologie der ASS-Intoleranz (NERD-Syndrom, M. Samter, M. Widal)

Die Pathophysiologie der NERD ist gekennzeichnet durch eine ausgeprägte Eosinophilie im Blut und im Gewebe [57] sowie durch ein Ungleichgewicht der Eicosanoide Cysteinyl-Leukotrien LTE4 und Prostaglandine PGD2 und PGE2, die über den Arachidonsäurestoffwechsel gebildet werden (Abb. 1). Cysteinyl-Leukotriene (LTC4, LTD4 und LTE4) sind potente Entzündungsmediatoren. Sie bewirken eine Verengung der Bronchien, eine erhöhte Gefäßdurchlässigkeit, Schleimproduktion und die Rekrutierung von Entzündungszellen. Arachidonsäure ist eine Fettsäure, die als Vorläufer für die Synthese verschiedener Eicosanoide, darunter Prostaglandine und Leukotriene, dient. Obwohl klinisch beobachtet und seit dem frühen 20. Jahrhundert bekannt, wurde der nichtallergische Pathomechanismus von Atemwegsreaktionen nach Einnahme von NSAID erstmals in den 1970er-Jahren von Szczeklik et al. beschrieben, indem sie die Hemmung der PG-Synthese nachgewiesen haben [103, 104]. Es besteht ein Ungleichgewicht zwischen den Cyclooxygenase-Isoformen 1 und 2 [80, 108]. Aspirin und andere COX-1-hemmende NSAID hemmen die COX-Enzyme, die für die Synthese von Prostaglandinen verantwortlich sind. Durch diese Hemmung wird das Ungleichgewicht weiter verschärft, was zu einer ausgeprägteren Entzündungsreaktion führt. Dies liegt wohl vor allem an der verminderten Synthese von entzündungshemmendem PGE2 und einer Überproduktion von entzündungsfördernden LTC4 und PGD2 [66].

In der Folge werden im Rahmen einer chronischen Entzündung Alarmine wie Interleukin-33 (IL-33) und thymisches stromales Lymphopoietin (TSLP) in der Atemwegsschleimhaut freigesetzt. Diese Zytokine aktivieren Th2-Lymphozyten und ILC2 zur Freisetzung von Zytokinen wie IL‑4, IL‑5 und IL-13, ein Prozess, der derzeit als T2-Entzündungsendotyp bekannt ist [57]. Von Mastzellen freigesetztes PGD2 ist ein wichtiger Effektor der T2-Immunantwort, die bei NERD-Patienten durch TSLP ausgelöst wird.

Thrombozyten werden bei NERD-Patienten aktiviert und setzen verschiedene Entzündungsmediatoren wie Thromboxan A2 und den thrombozytenaktivierenden Faktor frei. Das Ungleichgewicht zwischen Th2-Zellen und regulatorischen Tregs führt dazu, dass Th2-Zellen Zytokine absondern, die eosinophile Entzündungen fördern, während Tregs dazu beitragen, übermäßige Immunreaktionen zu unterdrücken. Der Umbau des Atemwegsepithels bei NERD geht über eine Becherzellmetaplasie hinaus und führt zu einem Netzwerk dysregulierter Entzündungszellen und epithelialer Mastzellveränderungen, die die T2-Entzündung aufrechterhalten. Die Dysregulation dieses angeborenen Systems trägt wesentlich zur Pathophysiologie der NERD bei [15].

## Hypersensitivitätsreaktion vom Typ VI – direkte zelluläre und entzündliche Reaktion auf chemische Substanzen

Die beschriebenen charakteristischen immunologischen Mechanismen haben zur Definition eines eigenen Hypersensitivitätstyps (Typ VI) für die direkten zellulären und entzündlichen Reaktionen auf chemische Substanzen wie NSAID geführt. Sie stellen somit Idiosynkrasiereaktionen mit Überempfindlichkeit auf nichtsteroidale Antirheumatika (NSAID) dar. Diese Reaktionen umfassen mindestens drei verschiedene Phänotypen, die vom Vorhandensein oder Fehlen einer zugrunde liegenden Atemwegs- oder Hauterkrankung abhängen: NSAID-exazerbierte Atemwegserkrankung bei Patienten mit Rhinitis und/oder Asthma mit oder ohne nasale Polyposis; NSAID-induzierte Hauterkrankung bei Patienten mit zugrunde liegender chronischer spontaner Urtikaria; und NSAID-induzierte akute Urtikaria/Angioödeme bei ansonsten gesunden Personen. In letzter Zeit wurden auch Phänotypen beschrieben, bei denen nach der Einnahme von NSAID gleichzeitig kutane und respiratorische Symptome auftreten. Der diesen Reaktionen zugrunde liegende Mechanismus wurde mit der Hemmung von COX‑1 und der Freisetzung von Eicosanoid-Mediatoren bei anfälligen Personen in Verbindung gebracht und auch für die NSAID-induzierte akute Urtikaria/Angioödeme beschrieben [38–41].

Weitere Beispiele für pharmakologische Wechselwirkungen sind Reaktionen, die durch G‑Protein-gekoppelte Rezeptoren (GPCR) in Mastzellen vermittelt werden. Vor Kurzem wurde ein neuer GPCR beschrieben, der als Mas-verwandter GPCR X2 (MRGPRX2) bekannt ist und unabhängig von Antikörpern durch eine Reihe von kationischen Liganden (Sekretagoga, „segregatogues“) aktiviert wird und vermutlich maßgeblich an anaphylaktoiden Reaktionen beteiligt ist. Zu diesen Liganden gehören entzündliche Peptide und Arzneimittel, die mit allergischen Reaktionen in Verbindung gebracht werden, wie z. B. nichtsteroidale neuromuskuläre Blocker (Atracurium, Mivacurium, Tubocurarin und Rocuronium) und Fluorchinolone (Ciprofloxacin, Levofloxacin, Moxifloxacin und Ofloxacin), neben anderen. Repräsentative Vertreter jeder bewerteten Arzneimittelgruppe führten zur Freisetzung von Histamin, Tumornekrosefaktor, Prostaglandin D2 (PGD2) und b‑Hexosaminidase aus LAD2-Zellen (menschlichen Mastzellen). Antimykotika, Aminoglykoside und Sulfonamide lösen über diesen Rezeptor eine Mastzelldegranulation aus. Darüber hinaus ist die Expression von MRGPRX2 in der Haut von Patienten mit schwerer chronischer Urtikaria erhöht.

## Diagnose der NERD

Anamnese und Provokationstests sind von zentraler Bedeutung für die Diagnose einer NERD [63, 105]. Kontrollierte Provokationstests bei Patienten mit Atemwegserkrankungen umfassen nasale, bronchiale oder orale Tests, während bei Aspirin-empfindlicher Urtikaria nur orale Provokationen sinnvoll sind [89]. Diese Tests müssen stets von erfahrenem Personal in spezialisierten Zentren durchgeführt werden, das in der Lage ist, schwere Reaktionen zu behandeln [97].

## Behandlungsoptionen für NERD

Die therapeutischen Optionen für NERD müssen auf die jeweilige klinische Entität eingehen: Asthma, CRSwNP, NSAID-Unverträglichkeit. Die Behandlung des Asthma bronchiale orientiert sich an den aktuellen Asthma-Leitlinien [58] und ist in der Regel als stufenweises Schema aufgebaut, wobei inhalative Kortikosteroide den Eckpfeiler bilden. Systemische Kortikosteroide sind als Notfallmedikamente auf schwere Exazerbationen begrenzt; die systemische Anwendung führt jedoch wahrscheinlich zu langfristigen Nebenwirkungen und sollte daher so weit wie möglich eingeschränkt werden.

Topisch-nasale Kortikosteroide sind die Standard-Basistherapie für die nasale Entzündung bei NERD [25, 71]. Die chronische Entzündung der oberen Atemwege bei NERD äußert sich typischerweise als CRS mit Nasenpolypen (CRSwNP). Bei Patienten mit NERD ändern weder der Verzicht auf NSAID noch topische Kortikosteroide oder Leukotrienantagonisten wesentlich den Krankheitsverlauf: Das Asthma bleibt auch bei optimaler Behandlung oft unkontrolliert, und die CRSwNP tritt trotz intranasaler und oraler Kortikosteroide und/oder mehrfacher endoskopischer Nasennebenhöhlenoperationen (ESS) [33] häufig rezidivierend auf oder verschlimmert sich. Ein chirurgischer Eingriff an den Nasennebenhöhlen ist die nichtpharmakologische Standardtherapie bei CRSwNP und begünstigt die Verteilung zukünftiger langfristiger topischer Nasensprays. Der Umfang des chirurgischen Eingriffs ist bei CRSwNP in der Regel sehr groß, da in allen Nebenhöhlen Polypengewebe abgetragen und entfernt werden muss. Eine multimodale Therapie einschließlich Operation scheint für eine begrenzte Zeit wirksamer zu sein [60]. Allerdings ist die Rezidivrate nach ESS bei NERD im Vergleich zu NSAID-toleranten Patienten sehr hoch [83]. Zweite oder mehrfache Operationen im Laufe der Zeit sind ein häufiges Phänomen bei NERD-Patienten.

Es gibt Hinweise auf positive Auswirkungen auf die unteren Atemwege, wenn die oberen Atemwege durch die Behandlung gut kontrolliert sind. Eine Metaanalyse mit einer Nachbeobachtungszeit von 26,4 Monaten ergab, dass die endoskopische Nasennebenhöhlenchirurgie für Asthmapatienten von Nutzen ist [112]. Zu den Verbesserungen gehörten eine bessere allgemeine Asthmakontrolle (76,1 % der Patienten), weniger Asthmaanfälle (84,8 %) und weniger Krankenhausaufenthalte (64,4 %). Der Einsatz von oralen Kortikosteroiden ging bei 72,8 % der Patienten zurück, auch der Einsatz von inhalativen Kortikosteroiden (28,5 %) und Bronchodilatatoren (36,3 %) nahm ab. Dieser Effekt wurde vor allem bei schwerem Asthma beobachtet.

Für schwere, unkontrollierte und/oder kortikosteroidabhängige Patienten stehen im Wesentlichen zwei pharmakologische Behandlungsmöglichkeiten zur Verfügung, die darauf abzielen, die Krankheit zu kontrollieren und den natürlichen Krankheitsverlauf zu verändern: Die adaptive Desaktivierungstherapie mit Aspirin/ASS (international bekannt als Aspirin-Therapie nach Desensibilisierung: ATAD) und die Behandlung mit Biologika. Diese Behandlungen werden oft als Alternativen vorgestellt, können aber auch sinnvoll kombiniert eingesetzt werden.

## Wirksamkeit der Aspirin-Therapie nach Desensibilisierung

Bislang wurde die ATAD klassischerweise mit dem Ansatz der kausalen Therapie bei Patienten mit schwerer rezidivierender CRSwNP und NERD durchgeführt [25, 42, 45, 90, 99–101, 105]. Ziel dieser Therapie ist es, durch die wiederholte Anwendung von ASS [47, 101, 106] eine Krankheitskontrolle und eine Stabilisierung der Pathophysiologie der Erkrankung zu erreichen.

Dieser therapeutische Ansatz geht auf die Beobachtungen von Zeiss und Lockey zurück, wonach bei NERD-Patienten nach der oralen Einnahme von Aspirin eine Refraktärphase auftritt. Nach der Einnahme von Aspirin gibt es einen Zeitraum, in dem eine erneute Einnahme nicht zu unerwünschten Wirkungen führt. Diese Refraktärphase kann zwischen 24 h und mehreren Tagen dauern [51] und kann durch tägliche Aspirineinnahme auf unbestimmte Zeit verlängert werden. Außerdem verbessert sich der klinische Zustand von Patienten mit NERD während dieser Refraktärphase: Stevenson et al., die als erste die ATAD [100] untersuchten, beobachteten nach sechs Monaten täglicher Aspirineinnahme einen Anstieg des FEV1, eine Verringerung des Bedarfs an oralen Kortikosteroiden und eine Verringerung der entzündlichen Schäden an der Nasenschleimhaut. Darüber hinaus führte die tägliche Einnahme von Aspirin zum Verschwinden der nasalen Symptome [100], aber es gab keine Bewertung der Größe der Nasenpolypen.

Die ATAD scheint in erster Linie über Verschiebungen im Verhältnis von PGD2/Cys-LTE4 [18, 62, 97] zu funktionieren. PGD2 steigt während der ATAD zunächst deutlich an [97]. Wenn dieser Anstieg jedoch sehr stark ist, sagt er weitere Reaktionen während des Desensibilisierungsprozesses voraus. Der anschließende Rückgang von PGD2 während der Erhaltungsphase mit täglicher Aspirineinnahme ist verbunden mit einer verringerten Expression von Cys-LT-Rezeptoren, entzündlichen Zellinfiltraten und verringerten Spiegeln von Typ-2-Zytokinen wie IL‑4, IL‑5 und IL-13, die von Th2-Lymphozyten freigesetzt werden [86]. Darüber hinaus führt ATAD zu einer verringerten Expression von LTE4 im Urin [2].

In den letzten 20 Jahren wurden Studien zu verschiedenen Applikationswegen veröffentlicht [76, 90, 100]. Derzeit hat sich die orale Verabreichung als Standardverfahren etabliert [99]. Es wurden mehrere andere Anwendungsprotokolle entwickelt, sowohl für die systemische als auch für die topische Verabreichung [94], darunter die intravenöse Verabreichung von ASS. Mit dem i.v.-Dosierungsschema wird eine kumulative Dosis von 900 mg i.v. in fünf Tagen Therapie erreicht. Ein Vorteil der intravenösen Verabreichung scheint die Möglichkeit zu sein, im Fall einer beginnenden Reaktion die Zufuhr der Substanz sofort zu unterbrechen, was nach oraler Verabreichung nicht möglich ist [78].

Die nasale Anwendung war vor allem bei der Behandlung von NERD in Verbindung mit CRSwNP wirksam. In einer prospektiven Studie über 5 Jahre konnte bei 43 NERD-Patienten gezeigt werden, dass die endonasale Applikation von Lysin-ASA in aufsteigender Dosierung (Dosissteigerung: wöchentlich 20 mg, 200 mg, 2000 mg, dann wöchentliche Erhaltungsdosis: 2000 mg) die Rezidivrate der nasalen Polyposis im Vergleich zu Placebo signifikant reduziert [76].

Eine kleine, verblindete Crossover-Studie mit 16 mg Lys-ASA führte nicht zu einer Verbesserung der Symptome, zeigte aber eine Verringerung der Leukotrienrezeptoren in der Nasenschleimhaut. Offene Studien weisen auf die Wirksamkeit dieser Applikationsform hin [69, 75], so konnte eine Verbesserung der Nasenatmung und des Riechvermögens nachgewiesen werden [77].

In einer anderen Studie wurde eine tägliche Erhaltungsdosis von nur 100 mg ASS gewählt, um mögliche Nebenwirkungen wie Gastritis zu minimieren. Über einen Zeitraum von drei Jahren wurde auch mit dieser deutlich niedrigeren Dosis ein klarer therapeutischer Effekt festgestellt, mit einer verringerten Wiederauftretensrate der Nasenpolyposis, einem geringeren Schweregrad des intrinsischen Asthmas und einer Verbesserung des Geruchssinns. Mit 100 mg ASS wurde jedoch kein signifikanter Unterschied im Nasenpolypen-Score zwischen der ASS-Gruppe und der Placebo-Gruppe nachgewiesen. Interessanterweise verbessert ATAD auch die Alkoholunverträglichkeit bei Patienten mit NERD [29].

Fruth et al. untersuchten 70 NERD-Patienten mit CRSwNP in einer randomisierten, doppelblinden, placebokontrollierten Studie. Aufgrund der hohen Abbrecherquote konnten nur 31 Patienten analysiert werden. Nach 36 Monaten traten in der Behandlungsgruppe seltener Nasenpolypen auf (primärer Endpunkt) (*p* = 0,0785), und der Nasenpolypen-Score war niedriger (*p* = 0,0702). Die Lebensqualität verbesserte sich (*p* = 0,0324), und die klinischen Symptome nahmen deutlich ab (*p* = 0,0083), und es wurden keine schweren aspirinbedingten Nebenwirkungen beobachtet. Auch wenn der primäre Endpunkt somit nicht mit statistischer Signifikanz erreicht wurde, führte ATAD mit einer Erhaltungsdosis von 100 mg in dieser Studie zu einer Verbesserung der klinischen Symptome und der Lebensqualität [28].

Es muss jedoch darauf hingewiesen werden, dass seit der ersten Vorstellung von ATAD durch Stevenson et al. [85] nur 5 doppelblinde, placebokontrollierte Studien zur Bewertung der Wirksamkeit und Sicherheit von ATAD durchgeführt wurden, an denen insgesamt nur 163 Patienten teilnahmen. Die geringe Zahl doppelblinder, placebokontrollierter Studien lässt sich durch die hohen regulatorischen Hürden für klinische Arzneimittelstudien und ein geringes Interesse der Hersteller von ASS-Präparaten sowie durch Schwierigkeiten bei der Verblindung der Patienten angesichts der mit Aspirin verbundenen häufigen Nebenwirkungen erklären.

### Therapie von CRSwNP und Asthma bei NERD mit Biologika

Da der T2-Endotyp bei etwa 80 % der Patienten mit CRSwNP in der westlichen Welt und insbesondere bei NERD-Patienten vorherrscht [87, 95, 98, 110], werden bei schwerer Erkrankung Biologika eingesetzt, die auf IL‑4, IL‑5, IL-13, TSLP und IgE abzielen [23, 34, 49]. Allgemeine Empfehlungen zur Diagnose und Behandlung von CRSwNP sind verfügbar [46], ebenso wie praxisorientierte Kriterien für den Einsatz der derzeit zugelassenen Biologika Dupilumab (Anti-IL4Rα), Omalizumab (Anti-IgE) und Mepolizumab (Anti-IL-5) [24, 26]. Biologika sind seit einiger Zeit in der Behandlung von Asthma bronchiale etabliert [11, 20, 59, 70, 84, 109].

Die Biologika Omalizumab, Mepolizumab und Dupilumab sind für den Einsatz bei Patienten mit schwerer, unkontrollierter CRSwNP als Zusatztherapie zu intranasalen Kortikosteroiden zugelassen [46, 48] und führen zu einer signifikanten Verringerung der Größe von Nasenpolypen, die stark genug ist, um möglicherweise eine signifikante Verringerung der Symptomlast, einschließlich Geruchsverlust, auch bei Patienten mit NERD zu erklären [34, 96]. Für Benralizumab wurden bei CRSwNP in einer Phase-III-Studie beide koprimären Endpunkte bei einem guten Sicherheitsprofil nur schwach erreicht [4], der Hersteller strebt hierfür keine Zulassung mehr in der EU an. Tezepelumab (Anti-TSLP) ist nicht nur für die Zusatzbehandlung von schwerem Asthma zugelassen [64]. Kürzlich wurden Ergebnisse einer Phase-III-Studie mit 408 Erwachsenen mit schwerer, unkontrollierter CRSwNP veröffentlicht. Die multizentrische Studie wurde an 112 Standorten in 10 Ländern durchgeführt [56]. 60 % der Teilnehmenden hatten Asthma, 70 % bereits eine Nasennebenhöhlenoperation, und 17 % eine Analgetikaunverträglichkeit. Trotz Symptomen unter standardisierter intranasaler Kortikosteroidtherapie erhielten die Teilnehmenden über 52 Wochen alle vier Wochen entweder Tezepelumab (210 mg) oder Placebo. Beide primären Endpunkte – Nasenpolypen-Score (−2,07) und Kongestions-Score (−1,03) – zeigten signifikante Verbesserungen, erste Effekte traten bereits nach wenigen Wochen auf. Sekundäre Endpunkte sprachen ebenfalls für Tezepelumab, darunter eine Verbesserung des SNOT-22 um 27,3 Punkte, besserer Geruchssinn, weniger Operationen (1 vs. 42) und geringerer Kortikosteroidgebrauch (5 vs. 36). Auch CT-Befunde (Lund-Mackay) und UPSIT-Werte verbesserten sich signifikant. Das Sicherheitsprofil entsprach dem von Placebo. Subgruppenanalysen deuten auf Wirksamkeit auch bei Patienten mit nichtklassischer Typ-2-Entzündung hin.

In den bisherigen Studien mit Biologika bei Patienten mit CRSwNP lag der Anteil der NERD-Patienten in den Mepolizumab-Studien bei 27 %, in den Dupilumab-Studien bei 28 %, in den Benralizumab-Studien bei 30 % und in den beiden Studien mit Omalizumab zwischen 17 und 39 % [9, 10, 27, 54, 55]. Es ist jedoch zu beachten, dass die Diagnose der NERD in all diesen Studien allein auf der Grundlage der Anamnese gestellt wurde, während für eine zuverlässige Diagnose der NERD eigentlich Provokationstests erforderlich sind [50]. Dennoch zeigen Post-hoc-Analysen der zulassungsrelevanten Studien, dass Patienten mit NERD von der Biologikatherapie in Bezug auf eine Verringerung der Polypengröße im Vergleich zur NSAID-toleranten Untergruppe gleich gut profitierten [6]. Darüber hinaus sprachen NERD-Patienten hinsichtlich der Verbesserung des Geruchssinns gleich gut auf die Dupilumab-Behandlung und hinsichtlich der nasalen Obstruktion auf die Benralizumab-Behandlung an. Dupilumab ist das einzige Biologikum, das einen Unterschied zwischen NSAID-toleranten und NSAID-intoleranten CRSwNP-Patienten zeigte: NERD-Patienten berichteten über eine signifikant größere Verbesserung der nasalen Obstruktion und des SNOT-22 [67]. Biomarker, die mit der Typ-2-Inflammation im Allgemeinen und der NERD im Besonderen in Zusammenhang stehen, verbesserten sich unter der Dupilumab-Behandlung ebenfalls [17].

Bei der Behandlung von Asthma ist die Wirksamkeit von Mepolizumab, Benralizumab und Dupilumab bei Patienten mit NERD gleichwertig, wenn sie richtig und nach den neuesten Behandlungsalgorithmen ausgewählt werden [54]. Es ist wichtig anzumerken, dass im Gegensatz zur Situation bei CRSwNP die Behandlung von Asthma in der Regel auf die Phäno- und Endotypisierung zugeschnitten werden kann.

Was den dritten Aspekt der Triade, die Aspirin‑/NSAID-Intoleranz, betrifft, so wurden in den letzten zehn Jahren zunächst vereinzelte Fallberichte und kleine Fallserien, dann aber auch größere Studien veröffentlicht, die über eine Aspirin-Verträglichkeit bei Patienten berichten, die mit Omalizumab oder Dupilumab behandelt wurden, und die diesen ansonsten eingeschränkten Patienten eine Tür in Bezug auf Schmerzlinderung und Thrombozytenaggregationshemmer öffnen [31, 32, 79]. Die Studie von Schneider et al. berichtete über eine heterogene Verbesserung der Aspirin-Verträglichkeit unter Dupilumab-Therapie, sodass allgemeine Annahmen und Empfehlungen für NERD-Patienten zum jetzigen Zeitpunkt riskant sind [91].

In einer kürzlich durchgeführten praxisnahen Studie bei Patienten mit schwerem Asthma und positivem oralem Provokationstest auf Acetylsalicylsäure zeigten Sánchez et al. eine Wirksamkeit von Omalizumab und Dupilumab, nicht aber von Mepolizumab und Benralizumab in Bezug auf das Ergebnis des oralen Provokationstests. Bei dieser Studie handelte es sich jedoch um eine kleine Pilotstudie, in die jeweils 9 Patienten unter Mepolizumab- und Benralizumab-Therapie und jeweils 10 Patienten unter Dupilumab- und Omalizumab-Therapie eingeschlossen wurden. Weitere Ergebnisse aus größeren Studien bleiben abzuwarten [88].

## Sicherheit und Voraussetzungen für eine ATAD oder Biologikatherapie

Langfristige Sicherheitsdaten für Biologika fehlen, insbesondere bei der Indikation CRSwNP [46]. Kurzfristig scheint der Einsatz von Biologika relativ sicher zu sein, mit einer Abbruchrate von weniger als 5 % in den meisten der veröffentlichten Phase-III-Studien [54]. In einer Metaanalyse von 24 randomisierten, kontrollierten Doppelblindstudien (RCT) war die Häufigkeit von unerwünschten Ereignissen bei den verschiedenen Biologika (Dupilumab, Omalizumab, Mepolizumab, Benralizumab und Reslizumab) vergleichbar und unterschied sich kaum von Placebo [72].

Ein häufig diskutiertes Problem bei der Behandlung mit Dupilumab ist das Risiko vorübergehend erhöhter Eosinophilenspiegel im Blut bei bis zu 10 % der Patienten. Obwohl dies in der Regel vorübergehend und unbedenklich ist, benötigten einige Patienten (< 5 %) eine Therapie, um die Behandlung mit Dupilumab gegen ihr Asthma oder CRSwNP fortzusetzen [54]. So war in einer Studie mit 1902 Patienten mit unkontrolliertem Asthma ein dauerhafter Abbruch der Dupilumab-Behandlung bei 7 Patienten erforderlich, im Vergleich zu einem Patienten in der Placebogruppe [19]. Eine Eosinophilie im peripheren Blut kann jedoch auch bei einer hochdosierten ASS-Therapie zur Behandlung von NERD auftreten [97].

Die Sicherheitsdaten zu hochdosiertem ATAD sind aufgrund der geringen Größe der Studien und der hohen Abbruchrate begrenzt, einige davon nicht behandlungsbedingt [53]. In einer retrospektiven Studie wurde die Rate schwerwiegender unerwünschter Wirkungen im Zusammenhang mit ATAD analysiert [107]. Am häufigsten führten gastrointestinale Blutungen (8,2 %), Anaphylaxie (0,92 %), Exazerbationen von Symptomen der oberen (0,92 %) und unteren Atemwege (3,7 %) und wiederkehrende Epistaxis (0,92 %) zum Abbruch der Behandlung.

Diese Ergebnisse stimmen mit anderen Berichten überein, in denen Gastritis als häufigster Grund für den Abbruch der Erhaltungstherapie von ATAD genannt wird [54, 68, 102]. Obwohl das Risiko von Blutungen bei langfristiger oraler ASS-Verabreichung nicht speziell bei NERD untersucht wurde, zeigen extrapolierte Daten aus der kardiovaskulären Anwendung von ASS ein um 50 % erhöhtes Risiko für gastrointestinale Blutungen [53, 97]. Das Risiko von ATAD in der Ära der Biologika wurde durch die Metaanalyse von Okhyman et al. hervorgehoben, die einen überzeugenden Risikounterschied zwischen Biologika und ATAD zeigte und somit die Wahl der Behandlung seither erheblich beeinflusst [72].

Eine alternative Option ist die Verwendung von topischem, intranasalem Lysin-Aspirin (Lys-ASA, die einzige lösliche Form) sowohl für nasale Provokationstests zur Diagnose einer NERD als auch für die anschließende Therapie. Bei intranasalem ATAD sind die verwendeten ASS-Dosen mit weniger als 100 mg niedriger als bei oralem ATAD, verursachen weniger Magenprobleme [35] und sind besser mit der kardiovaskulären Prophylaxe vereinbar, da orale Dosen über 100 mg (wie bei oralem ATAD üblich) mit schlechteren kardiovaskulären Ergebnissen verbunden sind.

Um eine akute pulmonale Beeinträchtigung zu verhindern, sind weitere Voraussetzungen für den Beginn der ATAD gegeben:

Es wird empfohlen, dass der Patient ein stabiles/kontrolliertes Asthma bronchiale mit einer akzeptablen Lungenfunktion (FEV_1_ > 70 %) haben sollte, bevor er mit der Therapie beginnt [54, 102], was in einigen Fällen schwierig oder unmöglich zu erreichen ist – daher sind Biologika die einzige logische, wirksame und sichere Option in dieser Untergruppe.

Während es bei schwerem Asthma klare Empfehlungen und einen Expertenkonsens zu den Indikationen und der Wirksamkeitsbewertung von Biologika gibt (GINA 2023, abrufbar unter: www.ginasthma.org, GEMA 5.3 www.gemasma.com), wurden für die CRSwNP noch keine international konsentierten Kriterien abschließend definiert. Zu den klinischen Kriterien, von denen einige oder alle vor Beginn einer Biologikatherapie erfüllt sein sollten, gehören die Diagnose eines bilateralen CRSwNP, die Rückverfolgbarkeit/der Nachweis einer Entzündung vom Typ 2, ein früherer Bedarf an systemischen Kortikosteroiden oder eine frühere Nasennebenhöhlenoperation, eine beeinträchtigte Lebensqualität, ein Versagen der Behandlung mit nasalen Kortikosteroiden, ein signifikanter Verlust des Geruchssinns und komorbides Asthma [5, 24]. Die Indikation sollte anhand einer standardisierten, validierten Checkliste überprüft werden. Bei NERD-Patienten ist Asthma jedoch nicht immer von Anfang an vorhanden und liegt möglicherweise zum Zeitpunkt der Diagnose der NERD noch nicht vor. Außerdem profitieren nicht alle NERD-Patienten letztendlich von einer ATAD [45], sodass Biologika hier eine Option bleiben.

## Faktoren, die die Wahl der pharmakologischen Behandlung beeinflussen

Biologika für NERD-bedingte Atemwegsentzündungen sind derzeit in der Europäischen Union, den Vereinigten Staaten, Kanada, Japan und Australien erhältlich. Derzeit gibt es keine eindeutigen Biomarker, die das Ansprechen eines Patienten auf eine Aspirindesensibilisierung oder eine Biologikatherapie vorhersagen können [115]. Die Zusatztherapie mit Biologika ist in Europa seit 2005 eine wichtige Option für schweres, unzureichend kontrolliertes Asthma und seit 2022 für CRSwNP. Die ATAD hingegen ist nur für Patienten mit Verdacht auf einen gestörten Arachidonsäure-Stoffwechsel im Zusammenhang mit NERD vorgesehen und daher auf diesen Endotyp beschränkt [53].

Komorbiditäten wie kardiovaskuläre oder rheumatische Erkrankungen können eine Entscheidung für eine ATAD begünstigen. Der NERD-Patient kann dann das prophylaktische ASS als Thrombozytenaggregationshemmer oder die täglichen NSAID als Analgetika einnehmen, um die mit einer rheumatischen Erkrankung verbundenen Schmerzen zu lindern [94] und gleichzeitig von einer ATAD profitieren; zu niedrig dosiertes ASS kann jedoch die CRSwNP und die Asthmakontrolle nicht verbessern.

Die ATAD ist jedoch nicht die bevorzugte Option, wenn absolute oder relative Kontraindikationen für ASS bestehen, wie z. B. eine peptische Ulkuskrankheit in der Vorgeschichte, eosinophile Ösophagitis (die auch mit NERD assoziiert sein kann), Niereninsuffizienz, Einnahme von Antikoagulanzien oder eine Koagulopathie in der Vorgeschichte [68, 102, 115]. Diese Patienten haben ein erhöhtes Risiko für ASS-bedingte Nebenwirkungen, da die langfristige Einnahme von ASS die Synthese von Magenprostaglandin (Prostazyklin oder PGI_2_) verringert und eine unzureichende Regeneration der Magenschleimhautzellen verursacht, was zu Magenschmerzen oder Geschwüren führen kann. Auch Blutungen können auftreten, da Aspirin die Funktion der Blutplättchen durch Acetylierung der Cyclooxygenase hemmt [114]. Zwischen 10 und 15 % der Patienten mit NERD können aufgrund von gastrointestinalen Nebenwirkungen oder Blutungen die Langzeitbehandlung mit ASS nicht fortsetzen [115]. Die Risiken und Komorbiditäten der oralen ASS-Gabe gewinnen auch bei älteren NERD-Patienten zunehmend an Bedeutung. Obwohl das Gesamtrisiko für Blutungen sehr gering ist, ist es deutlich höher als bei jüngeren Patienten und kann zu einer höheren Gesamtmortalität führen [53, 97]. Umgekehrt fehlt es an spezifischen Daten für Biologika bei älteren Menschen [46].

Bei schwangeren NERD-Patientinnen kann die Einnahme von Aspirin in einer Dosis von mehr als 81 mg täglich zu einem vorzeitigen Verschluss des Ductus arteriosus beitragen und das Risiko mütterlicher und fetaler Blutungen erhöhen. Daher sollte die orale Einnahme von ASS während der Schwangerschaft nicht begonnen oder fortgesetzt werden [102]. Andererseits sind diese Kontraindikationen für die orale Verabreichung von ASS im Rahmen der ATAD (Schwangerschaft, geplante Operationen wie z. B. eine Nasennebenhöhlenoperation) nur vorübergehend, und die Therapie kann danach nach einem erneuten Desensibilisierungsprozess fortgesetzt werden.

White et al. beschreiben drei häufige Situationen, in denen das Timing zwischen ATAD, Biologika und Operation eine entscheidende Rolle spielt [115]:

1. Ein Patient mit neu diagnostizierter Erkrankung sollte auf alle betroffenen Organdysfunktionen untersucht werden und eine angemessene Therapie erhalten, um die Krankheit in jeder Hinsicht zu kontrollieren. Eine chirurgische Beurteilung sollte nach einer CT-Untersuchung der Nasennebenhöhlen erfolgen, und eine sorgfältige Bewertung im Hinblick auf eine mögliche Behandlungseskalation sollte sich anschließen. In der Regel folgt eine Nasennebenhöhlenoperation mit/ohne ATAD; in ausgewählten Fällen kann jedoch auch eine Biologikatherapie infrage kommen. In der Publikation wird die Operation als „Debulking“ bezeichnet, wobei eine solche Polypektomie nicht längerfristig erfolgversprechend ist, sodass eher eine ESS durchgeführt werden sollte.
2. Der Patient mit einem frühen Polypenrezidiv ist ein idealer Kandidat für eine ATAD, da eine Aspirintherapie bei Patienten mit kürzlich erfolgter ESS aufgrund einer veränderten Reaktionsschwere auf ASS nach einer Sinusoperation [37] die beste Wirkung haben kann.
3. Bei anhaltendem Versagen der chirurgischen Maßnahmen sollten die Risiken und der Nutzen von ATAD oder eines Biologikums unter Berücksichtigung der Präferenzen des Patienten, bestehender Komorbiditäten wie kardiovaskulärer Erkrankungen oder der Notwendigkeit einer Behandlung mit NSAID oder eines bevorstehenden ESS erörtert werden. In ähnlicher Weise schlugen Buchheit et al. einen Behandlungsalgorithmus vor, bei dem Biologika erst nach vollständiger ESS und einem Versuch der Aspirindesensibilisierung angeboten werden, da die Kosten für Biologika hoch sind, und es an klaren Daten zur langfristigen Sicherheit mangelt [16].

Abgesehen von den medizinischen Gründen, die für die eine oder andere Behandlung sprechen, sind die Erwartungen des einzelnen Patienten, die Wahrscheinlichkeit der Therapietreue und die subjektive Bewertung des Behandlungsschemas in der NERD-orientierten Behandlung zu berücksichtigen. Patienten, die bei einer ATAD ihre ASS-Dosis für mehr als 3–7 Tage auslassen, werden allmählich wieder intolerant und müssen dann erneut mit ASS desensibilisiert werden [54, 97]. Zudem ist zu erwarten, dass die langfristige Therapietreue bei Medikamenten, die zu Nebenwirkungen neigen, geringer ist, wie in früheren Studien beobachtet wurde [28]. Darüber hinaus wurden die Patienten gebeten, ihre bisherigen Behandlungserfahrungen retrospektiv zu bewerten. Die Patienten wurden nach der Wirksamkeit und der Gesamtbewertung von entweder ATAD + Operation vs. Biologika oder ATAD vs. Biologika + ATAD befragt und bevorzugten die Biologikatherapie in Bezug auf Wirksamkeit, Wohlbefinden und Dauer der Krankheitskontrolle [9, 96]. Im Rahmen eines patientenzentrierten Ansatzes sollten diese Erfahrungen auch in der Gesamtbewertung berücksichtigt werden und in künftigen Studienansätzen strukturierter behandelt werden.

## Kombination von gleichzeitiger ATAD und Biologika

In einer Studie am Brigham and Women’s Boston Hospital berichteten 24 von 98 Patienten über die gleichzeitige Verwendung von ATAD und einem Biologikum [68]. In der Tat muss die Wahl nicht unbedingt dichotom sein. Mehr noch, ATAD und T2-Biologika können einen synergistischen Effekt haben, wenn sie in Kombination eingesetzt werden. T2-Biologika helfen ausreichend in schwierigen Fällen, in denen das Ansprechen auf eine ATAD nur teilweise ist, aber nicht ausreicht, um die Notwendigkeit einer weiteren Therapie auszuschließen. Insbesondere in der Anfangsphase einer ATAD (Induktionsphase) können T2-Biologika auch eine Verschlechterung der klinischen Manifestationen der NERD verhindern und damit die ATAD überhaupt erst ermöglichen.

Insbesondere NERD-Patienten mit schwerem Asthma bronchiale oder ausgeprägter Urtikaria aufgrund einer manifesten Überproduktion von PGD2 und LTE4 benötigen möglicherweise die Zugabe eines T2-Biologikums, um eine tägliche NSAID-Dosis im Rahmen einer ATAD zu vertragen [12]. Einige Daten deuten darauf hin, dass die Wirkung von T2-Biologika über die Vorbeugung exazerbierter Reaktionen hinausgeht und das Potenzial hat, eine NSAID-Toleranz zu induzieren: Von 33 Patienten, die sich vor und nach 6 Monaten Anti-IgE-Therapie mit Omalizumab einem ASS-Provokationstest unterzogen, entwickelten 56 % eine vollständige NSAID-Toleranz [81]. In einer anderen Studie wurde die Wirksamkeit einer 6‑monatigen Behandlung mit Dupilumab bei der Herbeiführung einer Toleranz gegenüber ASS nachgewiesen [91]. Außerdem wurde bei CRSwNP-Patienten nach der Behandlung mit Dupilumab eine Verringerung der LT festgestellt [55]. Die Induktion einer Toleranz gegenüber höheren NSAID-Dosierungen muss in Zukunft bei der Bewertung des Gesamtbehandlungserfolgs genauer untersucht werden.

## Fazit für die Praxis

- Die Verbreitung und eine rasche Akzeptanz der neuen Nomenklatur für die CRSwNP sind für die Entwicklung des gesamten Fachgebiets von entscheidender Bedeutung.
- Der neue Ansatz, der auf Krankheitsmechanismen und Endotypen statt auf Phänotypen basiert, kann zur Entwicklung neuer Diagnoseinstrumente, verbesserter therapeutischer Strategien und eines besseren Krankheitsmanagements führen sowie die künftige translationale und klinische Forschung zu innovativeren Strategien anleiten.
- Hierzu könnten therapeutisch unter anderem neue Biologika und Strategien zur Veränderung des Mikrobioms gehören.
- Zu den wichtigsten Entdeckungen, die in dieser neuen Systematik berücksichtigt werden, gehören vor allem die Rolle von T‑Zell-Untergruppen, die Rolle angeborener Immunzellen, die Rolle von Nicht-T2-Reaktionen beim Gewebeumbau und bei der Chronifizierung, die Mechanismen virusbedingter Exazerbationen, die Rolle der Epithelbarriere, Fortschritte beim Immunstoffwechsel und seine Auswirkungen auf die Immunpolarisierung sowie neuroimmunogene Mechanismen, die zu chronischen Entzündungsreaktionen beitragen.
- Unseres Erachtens liegt der Hauptvorteil dieser auf der Immunreaktion basierenden Nomenklatur in einem nuancierteren Konzept, das Möglichkeiten der Präzisionsmedizin und personalisierten Medizin eröffnet.
- Das endgültige Ziel besteht darin, die Behandlung auf den einzelnen Patienten zuzuschneiden, und zwar auf der Grundlage spezifischer Immunreaktionen und Analysen von Exposom und Metaexposom.
- Wir haben daher in Teil 1 dieser Serie die Grundlagen der neuen Nomenklatur, die Hypersensitivitätsreaktionen vom Typ IVa–c als Korrelat zu T1-, T2- und T3-Endotypen erörtert.
- Teil 2 umfasste die epithelialen Barrieredefekte (Hypersensitivitätsreaktionen vom Typ V).
- Der vorliegende Teil 3 schließt die Reihe ab durch Darstellung der direkten zellulären und entzündlichen Reaktion auf chemische Substanzen (Typ VI).

## Abkürzungen

APC: Antigenpräsentierende Zellen
ASS: Acetylsalicylsäure
ATAD: Adaptive Desaktivierungstherapie mit ASS
COX: Cyclooxygenase
CRS: Chronische Rhinosinusitis
CRSsNP: Chronische Rhinosinusitis ohne Nasenpolypen
CRSwNP: Chronische Rhinosinusitis mit Nasenpolypen
Cys-LT: Cysteinyl-Leukotriene
DC: Dendritische Zellen
EAACI: Europäische Akademie für Allergie und klinische Immunologie
ECP: Eosinophiles kationisches Protein
ESS: Endoskopische Nasennebenhöhlenchirurgie
HLA: Humanes Leukozytenantigen
IFN: Interferon
Ig: Immunglobulin
IL: Interleukin
ILC: Angeborene lymphoide Zellen
LTE4: Leukotrien E4
NERD: „NSAID-exacerbated respiratory disease“
NSAID: „Nonsteroidal anti-inflammatory drug“
PGD2: Prostaglandin D2
PGE2: Prostaglandin E2
ROS: Reaktive Sauerstoffspezies
T2: Typ-2-Immunreaktion
Th: T‑Helfer-Lymphozyten
TH2: T‑Helfer-Zellen des Typs 2
TNF: Tumornekrosefaktor
TSLP: Thymisches stromales Lymphopoietin, („thymic stromal lymphopoietin“)

## References

[1] Akdis CA, Bachert C, Cingi C et al (2013) Endotypes and phenotypes of chronic rhinosinusitis: a PRACTALL document of the European Academy of Allergy and Clinical Immunology and the American Academy of Allergy, Asthma & Immunology. The Journal of allergy and clinical immunology 131:1479–1490 10.1016/j.jaci.2013.02.036 PMC4161279 23587334

[2] Arm JP, O’hickey SP, Spur BW et al (1989) Airway responsiveness to histamine and leukotriene E4 in subjects with aspirin-induced asthma. The American review of respiratory disease 140:148–153 10.1164/ajrccm/140.1.148 2546469

[3] Arshi S, Eslami N, Nabavi M et al (2020) Aspirin Sensitivity in Patients with Moderate to Severe Asthma. Iranian journal of allergy, asthma, and immunology 19:447–451 10.18502/ijaai.v19i4.4120 33463111

[4] Bachert C, Han JK, Desrosiers MY et al (2021) Efficacy and safety of benralizumab in chronic rhinosinusitis with nasal polyps: A randomized, placebo-controlled trial. The Journal of allergy and clinical immunology 149:1309–1317.e1312 10.1016/j.jaci.2021.08.030 34599979

[5] Bachert C, Han JK, Wagenmann M et al (2020) EUFOREA expert board meeting on uncontrolled severe chronic rhinosinusitis with nasal polyps (CRSwNP) and biologics: Definitions and management. The Journal of allergy and clinical immunology 147:29–36 10.1016/j.jaci.2020.11.013 33227318

[6] Bachert C, Sousa AR, Han JK et al (2022) Mepolizumab for chronic rhinosinusitis with nasal polyps: Treatment efficacy by comorbidity and blood eosinophil count. The Journal of allergy and clinical immunology 149:1711–1721.e1716 10.1016/j.jaci.2021.10.040 35007624

[7] Bergmann KC (2022) Biology of house dust mites and storage mites. Allergo Journal International 31:272–278

[8] Bergmann KC (2022) Frequency of sensitizations and allergies to house dust mites. Allergo Journal International 31:279–283

[9] Bertlich M, Freytag S, Dombrowski T et al (2022) Subgroups in the treatment of nasal polyposis with dupilumab: A retrospective study. Medicine 101:e31031 10.1097/MD.0000000000031031 PMC9666224 36397403

[10] Bertlich M, Ihler F, Bertlich I et al (2021) Management of chronic rhinosinusitis with nasal polyps in Samter triad by low-dose ASA desensitization or dupilumab. Medicine 100:e27471 10.1097/MD.0000000000027471 PMC8500659 34622875

[11] Bölke G, Church MK, Bergmann KC (2019) Comparison of extended intervals and dose reduction of omalizumab for asthma control. Allergo Journal International 28:1–4

[12] Bosso JV (2020) Aspirin desensitization for aspirin-exacerbated respiratory disease in the era of biologics: Clinical perspective. International forum of allergy & rhinology 11:822–823 10.1002/alr.22709 33070455

[13] Brehler R (2023) Clinic and diagnostics of house dust mite allergy. Allergo Journal International 32:1–4

[14] Brehler R, Klimek L (2023) Allergen characteristics, quality, major allergen content and galenics for mite allergen-specific immunotherapy preparations. Allergo Journal International 32:5–9

[15] Buchheit KM, Cahill KN, Katz HR et al. (2015) Thymic stromal lymphopoietin controls prostaglandin D2 generation in patients with aspirin-exacerbated respiratory disease. The Journal of allergy and clinical immunology 137:1566–1576.e1565 10.1016/j.jaci.2015.10.020 PMC4860132 26691435

[16] Buchheit KM, Laidlaw TM, Levy JM (2021) Immunology-based recommendations for available and upcoming biologics in aspirin-exacerbated respiratory disease. The Journal of allergy and clinical immunology 148:348–350 10.1016/j.jaci.2021.06.019 PMC9022378 34174296

[17] Buchheit KM, Sohail A, Hacker J et al. (2022) Rapid and sustained effect of dupilumab on clinical and mechanistic outcomes in aspirin-exacerbated respiratory disease. J Allergy Clin Immunol 150:415–424 10.1016/j.jaci.2022.04.007 PMC9378638 35460728

[18] Cahill KN, Cui J, Kothari P et al. (2018) Unique Effect of Aspirin Therapy on Biomarkers in Aspirin-exacerbated Respiratory Disease. A Prospective Trial. American journal of respiratory and critical care medicine 200:704–711 10.1164/rccm.201809-1755OC PMC6775876 30978291

[19] Castro M, Corren J, Pavord ID et al. (2018) Dupilumab Efficacy and Safety in Moderate-to-Severe Uncontrolled Asthma. The New England journal of medicine 378:2486–2496 10.1056/NEJMoa1804092 29782217

[20] Ciprandi G, Schiavetti I, Ricciardolo FLM (2023) Patients with asthma consulting an allergist differ from those consulting a pulmonologist. Allergo Journal International

[21] Coombs PRaG, Gell PG (1968) Classification of Allergic Reactions Responsible for Clinical Hypersensitivity and Disease. In: Gell RR (ed) Clinical Aspects of Immunology. Oxford University Press, Oxford, pp 575–596

[22] Cuevas M, Polk ML, Becker S et al (2022) Rhinitis allergica in storage mite allergy. Allergo Journal International 31:59–68

[23] De Prins L, Raap U, Mueller T et al (2022) White Paper on European Patient Needs and Suggestions on Chronic Type 2 Inflammation of Airways and Skin by EUFOREA. Frontiers in allergy 3:889221 10.3389/falgy.2022.889221 PMC9234878 35769567

[24] Fokkens WJ, Lund V, Bachert C et al (2019) EUFOREA consensus on biologics for CRSwNP with or without asthma. Allergy 74:2312–2319 10.1111/all.13875 PMC6972984 31090937

[25] Fokkens WJ, Lund VJ, Hopkins C et al (2020) European Position Paper on Rhinosinusitis and Nasal Polyps 2020. Rhinology 58:1–464 10.4193/Rhin20.600 32077450

[26] Fokkens WJ, Viskens AS, Backer V et al (2023) EPOS/EUFOREA update on indication and evaluation of Biologics in Chronic Rhinosinusitis with Nasal Polyps 2023. Rhinology 61:194–202 10.4193/Rhin22.489 36999780

[27] Forster-Ruhrmann U, Stergioudi D, Pierchalla G et al (2020) Omalizumab in patients with NSAIDs-exacerbated respiratory disease. Rhinology 58:226–232 10.4193/Rhin19.318 32077449

[28] Fruth K, Pogorzelski B, Schmidtmann I et al (2013) Low-dose aspirin desensitization in individuals with aspirin-exacerbated respiratory disease. Allergy 68:659–665 10.1111/all.12131 23464577

[29] Glicksman JT, Parasher AK, Doghramji L et al (2018) Alcohol-induced respiratory symptoms improve after aspirin desensitization in patients with aspirin-exacerbated respiratory disease. International forum of allergy & rhinology 8:1093–1097 10.1002/alr.22168 30007020

[30] Grosse-Kathoefer S, Aglas L, Ferreira F et al (2023) What inhalant allergens can do and not do?—The cooperation of allergens and their source in Th2 polarization and allergic sensitization. Allergo Journal International 32:258–268

[31] Guillén D, Bobolea I, Calderon O et al (2015) Aspirin desensitization achieved after omalizumab treatment in a patient with aspirin-exacerbated urticaria and respiratory disease. J Investig Allergol Clin Immunol 25:133–135 25997307

[32] Hayashi H, Mitsui C, Nakatani E et al (2016) Omalizumab reduces cysteinyl leukotriene and 9α,11β-prostaglandin F2 overproduction in aspirin-exacerbated respiratory disease. J Allergy Clin Immunol 137:1585–1587 10.1016/j.jaci.2015.09.034 26559322

[33] Hellings PW (2022) SWOT Analysis of Chronic Rhinosinusitis Care Anno 2022. The journal of allergy and clinical immunology. In practice 10:1468–1471 10.1016/j.jaip.2022.01.039 35131515

[34] Hellings PW, Verhoeven E, Fokkens WJ (2021) State-of-the-art overview on biological treatment for CRSwNP. Rhinology 59:151–163 10.4193/Rhin20.570 33459728

[35] Howe R, Mirakian RM, Pillai P et al (2014) Audit of nasal lysine aspirin therapy in recalcitrant aspirin exacerbated respiratory disease. The World Allergy Organization journal 7:18 10.1186/1939-4551-7-18 PMC4114086 25097720

[36] Jerschow E, Dubin R, Chen CC et al (2024) Aspirin-exacerbated respiratory disease is associated with variants in filaggrin, epithelial integrity, and cellular interactions. The journal of allergy and clinical immunology. Global 3:100205 10.1016/j.jacig.2024.100205 PMC10838899 38317805

[37] Jerschow E, Edin ML, Chi Y et al (2018) Sinus Surgery Is Associated with a Decrease in Aspirin-Induced Reaction Severity in Patients with Aspirin Exacerbated Respiratory Disease. The journal of allergy and clinical immunology. In practice 7:1580–1588 10.1016/j.jaip.2018.12.014 PMC6511299 30580047

[38] Jutel M (2024) Neue Nomenklatur allergischer Erkrankungen nach EAACI-Standard: Teil 3: Nomenklatur von Allergien vom Typ 4 - Executive Summary eines EAACI-Positionspapiers. Allergo Journal 33:16–27

[39] Jutel M, Agache I, Zemelka-Wiacek M et al (2023) Nomenclature of allergic diseases and hypersensitivity reactions: Adapted to modern needs: An EAACI position paper [Correction: Jan. 2024, 79(1), p. 269–273]. Allergy 78(11):2851–2874 10.1111/all.15889 37814905

[40] Jutel MAI, Ollert M, Vieths S, Schwarze J, Akdis Ca, Klimek L (2024) Neue Nomenklatur allergischer Erkrankungen nach EAACI-Standard: Teil 2: Nomenklatur von Allergien vom Typ 2 und 3 - Executive Summary eines EAACI-Positionspapiers. Allergo Journal 33:16–24

[41] Jutel M, Ollert M, Vieths S et al (2024) Neue Nomenklatur allergischer Erkrankungen nach EAACI-Standard: Teil 1: Grundlagen und Nomenklatur von Soforttyp-Allergien (Typ 1) - Executive Summary eines EAACI-Positionspapiers. Allergo Journal 33:16–25

[42] Kirsche H, Klimek L (2015) ASS-Intoleranz-Syndrom und persistierende Rhinosinusitis : Differentialdiagnostik und Therapie. Hno 63:357–363 10.1007/s00106-015-0008-7 25929893

[43] Klimek L, Brehler R, Bergmann KC et al (2023) Avoidance measures for mite allergy—an update. Allergo Journal International 32:18–27

[44] Klimek L, Brehler R, Casper I et al (2023) Allergen immunotherapy in house dust mite-associated allergic rhinitis: efficacy of the 300 IR mite tablet. Allergo Journal International 32:10–17

[45] Klimek L, Dollner R, Pfaar O et al (2014) Aspirin desensitization: useful treatment for chronic rhinosinusitis with nasal polyps (CRSwNP) in aspirin-exacerbated respiratory disease (AERD)? Current allergy and asthma reports 14:441 10.1007/s11882-014-0441-9 24682773

[46] Klimek L, Förster-Ruhrmann U, Beule AG et al (2022) Indicating biologics for chronic rhinosinusitis with nasal polyps (CRSwNP). Allergo J Int 31:149–160

[47] Klimek L, Pfaar O (2009) Aspirin intolerance: does desensitization alter the course of the disease? Immunology and allergy clinics of North America 29:669–675 10.1016/j.iac.2009.07.008 19879442

[48] Koennecke M, Klimek L, Mullol J et al (2018) Subtyping of polyposis nasi: phenotypes, endotypes and comorbidities. Allergo journal international 27:56–65 10.1007/s40629-017-0048-5 PMC5842507 29564208

[49] Kolkhir P, Akdis CA, Akdis M et al (2023) Type 2 chronic inflammatory diseases: targets, therapies and unmet needs. Nature reviews. Drug discovery 22:743–767 10.1038/s41573-023-00750-1 37528191

[50] Kowalski ML, Agache I, Bavbek S et al (2019) Diagnosis and management of NSAID-Exacerbated Respiratory Disease (N-ERD)-a EAACI position paper. Allergy 74:28–39 10.1111/all.13599 30216468

[51] Kowalski ML, Grzelewska-Rzymowska I, Rozniecki J et al (1984) Aspirin tolerance induced in aspirin-sensitive asthmatics. Allergy 39:171–178 10.1111/j.1398-9995.1984.tb02621.x 6711770

[52] Kowalski ML, Makowska JS (2015) Seven steps to the diagnosis of NSAIDs hypersensitivity: how to apply a new classification in real practice? Allergy, asthma & immunology research 7:312–320 10.4168/aair.2015.7.4.312 PMC4446629 25749768

[53] Laidlaw TM (2020) Aspirin desensitization vs biologics for patients with aspirin-exacerbated respiratory disease. Annals of allergy, asthma & immunology : official publication of the American College of Allergy, Asthma, & Immunology 126:118–119 10.1016/j.anai.2020.08.008 32771353

[54] Laidlaw TM, Chu DK, Stevens WW et al (2022) Controversies in Allergy: Aspirin Desensitization or Biologics for Aspirin-Exacerbated Respiratory Disease-How to Choose. The journal of allergy and clinical immunology. In practice 10:1462–1467 10.1016/j.jaip.2021.12.030 34999274

[55] Laidlaw TM, Mullol J, Fan C et al (2019) Dupilumab improves nasal polyp burden and asthma control in patients with CRSwNP and AERD. The journal of allergy and clinical immunology. In practice 7:2462–2465.e2461 10.1016/j.jaip.2019.03.044 30954643

[56] Lipworth BJ, Han JK, Desrosiers M et al. (2025) Tezepelumab in Adults with Severe Chronic Rhinosinusitis with Nasal Polyps. N Engl J Med 10.1056/NEJMoa2414482 40106374

[57] Liu T, Kanaoka Y, Barrett NA et al (2015) Aspirin-Exacerbated Respiratory Disease Involves a Cysteinyl Leukotriene-Driven IL-33-Mediated Mast Cell Activation Pathway. Journal of immunology (Baltimore, Md. : 1950) 195:3537–3545 10.4049/jimmunol.1500905 PMC4592820 26342029

[58] Lommatzsch M, Criée C‑P, De Jong CCM et al. (2023) S2k-Leitlinie zur fachärztlichen Diagnostik und Therapie von Asthma 2023. Pneumologie (Stuttgart, Germany) 10.1055/a-2070-2135 37406667

[59] Lommatzsch M, Stoll P (2016) Novel strategies for the treatment of asthma. Allergo journal international 25:11–17 10.1007/s40629-016-0093-5 PMC4792349 27069845

[60] Lourijsen ES, Reitsma S, Vleming M et al (2022) Endoscopic sinus surgery with medical therapy versus medical therapy for chronic rhinosinusitis with nasal polyps: a multicentre, randomised, controlled trial. Lancet Respir Med 10:337–346 10.1016/S2213-2600(21)00457-4 35012708

[61] Luperto P, Masieri S, Cavaliere C et al (2022) Nasal cytology identifies allergic rhinitis phenotypes for managing allergen immunotherapy in clinical practice. Allergo Journal International 31:51–55

[62] Lyly A, Laidlaw TM, Lundberg M (2021) Pathomechanisms of AERD-Recent Advances. Frontiers in allergy 2:734733 10.3389/falgy.2021.734733 PMC8974777 35387030

[63] Mailing HJ, Weeke B (1993) Position Paper: Immunotherapy. Allergy 48:9–35 10.1111/j.1398-9995.1993.tb04754.x 8342741

[64] Menzies-Gow A, Corren J, Bourdin A et al (2021) Tezepelumab in Adults and Adolescents with Severe, Uncontrolled Asthma. N Engl J Med 384:1800–1809 10.1056/NEJMoa2034975 33979488

[65] Mülleneisen N, Springob M, Salge S et al (2022) The long road from the first symptoms of mite allergy to mite-specific immunotherapy. Allergo Journal International 31:284–287

[66] Mullol J, Boyce J, Dahlén SE et al (2021) Eicosanoid dysregulation and type 2 inflammation in AERD. J Allergy Clin Immunol 148:1157–1160 10.1016/j.jaci.2021.08.015 34464635

[67] Mullol J, Laidlaw TM, Bachert C et al (2022) Efficacy and safety of dupilumab in patients with uncontrolled severe chronic rhinosinusitis with nasal polyps and a clinical diagnosis of NSAID-ERD: Results from two randomized placebo-controlled phase 3 trials. Allergy 77:1231–1244 10.1111/all.15067 PMC9292324 34459002

[68] Mullur J, Steger CM, Gakpo D et al (2022) Aspirin desensitization and biologics in aspirin-exacerbated respiratory disease: Efficacy, tolerability, and patient experience. Annals of allergy, asthma & immunology : official publication of the American College of Allergy, Asthma, & Immunology 128:575–582 10.1016/j.anai.2022.01.043 PMC9058196 35131410

[69] Nucera E, Schiavino D, Milani A et al (2000) Effects of lysine-acetylsalicylate (LAS) treatment in nasal polyposis: two controlled long term prospective follow up studies. Thorax 55(Suppl 2):S75–S78 10.1136/thorax.55.suppl_2.S75 PMC1765967 10992567

[70] Ong KY (2019) What’s new in the Global Initiative for Asthma 2018 report and beyond. Allergo Journal International 28:63–72

[71] Orlandi RR, Kingdom TT, Smith TL et al (2021) International consensus statement on allergy and rhinology: rhinosinusitis 2021. International forum of allergy & rhinology 11:213–739 10.1002/alr.22741 33236525

[72] Oykhman P, Paramo FA, Bousquet J et al (2021) Comparative efficacy and safety of monoclonal antibodies and aspirin desensitization for chronic rhinosinusitis with nasal polyposis: A systematic review and network meta-analysis. The Journal of allergy and clinical immunology 149:1286–1295 10.1016/j.jaci.2021.09.009 34543652

[73] Papadopoulos NG, Bernstein JA, Demoly P et al (2015) Phenotypes and endotypes of rhinitis and their impact on management: a PRACTALL report. Allergy 70:474–494 10.1111/all.12573 25620381

[74] Papadopoulos NG, Guibas GV (2016) Rhinitis Subtypes, Endotypes, and Definitions. Immunology and allergy clinics of North America 36:215–233 10.1016/j.iac.2015.12.001 PMC7127043 27083098

[75] Parikh A, Scadding GK (2014) Topical nasal lysine aspirin in aspirin-sensitive and aspirin-tolerant chronic rhinosinusitis with nasal polyposis. Expert review of clinical immunology 10:657–665 10.1586/1744666X.2014.901889 24684687

[76] Parikh AA, Scadding GK (2005) Intranasal lysine-aspirin in aspirin-sensitive nasal polyposis: a controlled trial. The Laryngoscope 115:1385–1390 10.1097/01.MLG.0000166702.38850.1B 16094110

[77] Pendolino AL, Scadding GK, Scarpa B et al (2021) A retrospective study on long-term efficacy of intranasal lysine-aspirin in controlling NSAID-exacerbated respiratory disease. European archives of oto-rhino-laryngology : official journal of the European Federation of Oto-Rhino-Laryngological Societies (EUFOS) : affiliated with the German Society for Oto-Rhino-Laryngology - Head and Neck Surgery 279:2473–2484 10.1007/s00405-021-07063-2 PMC8986745 34480600

[78] Pfaar O, Klimek L (2006) Aspirin desensitization in aspirin intolerance: update on current standards and recent improvements. Current opinion in allergy and clinical immunology 6:161–166 10.1097/01.all.0000225153.45027.6a 16670507

[79] Phillips-Angles E, Barranco P, Lluch-Bernal M et al (2017) Aspirin tolerance in patients with nonsteroidal anti-inflammatory drug-exacerbated respiratory disease following treatment with omalizumab. The journal of allergy and clinical immunology. In practice 5:842–845 10.1016/j.jaip.2016.12.013 28117272

[80] Picado C, Fernandez-Morata JC, Juan M et al (1999) Cyclooxygenase‑2 mRNA is downexpressed in nasal polyps from aspirin-sensitive asthmatics. Am J Respir Crit Care Med 160:291–296 10.1164/ajrccm.160.1.9808048 10390414

[81] Quint T, Dahm V, Ramazanova D et al (2021) Omalizumab-Induced Aspirin Tolerance in Nonsteroidal Anti-Inflammatory Drug-Exacerbated Respiratory Disease Patients Is Independent of Atopic Sensitization. The journal of allergy and clinical immunology. In practice 10:506–516.e506 10.1016/j.jaip.2021.09.050 34678497

[82] Rajan JP, Wineinger NE, Stevenson DD et al (2015) Prevalence of aspirin-exacerbated respiratory disease among asthmatic patients: A meta-analysis of the literature. The Journal of allergy and clinical immunology 135:676–681.e671 10.1016/j.jaci.2014.08.020 25282015

[83] Rodriguez-Van Strahlen C, Arancibia C, Calvo-Henriquez C et al. (2024) Systematic Review of Long Term Sinonasal Outcomes in CRSwNP after Endoscopic Sinus Surgery: A call for Unified and Standardized Criteria and Terms. Current allergy and asthma reports 10.1007/s11882-024-01154-w PMC11297087 38913122

[84] Rothe T (2018) A century of “intrinsic asthma” - a view on the development of phenotyping in asthma in the last 100 years. Allergo Journal International 27:215–219

[85] Samter M, Beers RF Jr (1967) Concerning the nature of intolerance to aspirin. J Allergy 40:281–293 10.1016/0021-8707(67)90076-7 5235203

[86] San Nicoló M, Högerle C, Gellrich D et al (2019) The time course of nasal cytokine secretion in patients with aspirin-exacerbated respiratory disease (AERD) undergoing aspirin desensitization: preliminary data. European archives of oto-rhino-laryngology : official journal of the European Federation of Oto-Rhino-Laryngological Societies (EUFOS) : affiliated with the German Society for Oto-Rhino-Laryngology - Head and Neck Surgery 277:445–452 10.1007/s00405-019-05704-1 31655881

[87] Sanchez-Collado I, Mora T, Munoz-Cano R et al (2022) Prevalence of Chronic Rhinosinusitis with Nasal Polyps in Catalonia (Spain): a retrospective, large-scale population-based study. Rhinology 60:384–396 10.4193/Rhin21.364 36150155

[88] Sánchez J, García E, Lopez JF et al (2023) Nonsteroidal Anti-inflammatory Drug (NSAID) Tolerance After Biological Therapy in Patients With NSAID-Exacerbated Respiratory Disease: A Randomized Comparative Trial. The journal of allergy and clinical immunology. In practice 11:2172–2179 10.1016/j.jaip.2023.04.033 37146885

[89] Schapowal A (1998) Pseudoallergische Reaktionen auf nicht steroidale Antiphlogistika. In: Heppt W, Bachert C (eds) Praktische Allergologie – Schwerpunkt HNO-Heilkunde. Thieme, Stuttgart, pp 233–238

[90] Schmitz-Schumann M, Schaub E, Virchow C (1982) Inhalative Provokation mit Lysin-Azetylsalizylsäure bei Analgetika-Asthme-Syndrom. Praxis und Klinik der Pneumologie 36:17–21 6808487

[91] Schneider S, Poglitsch K, Morgenstern C et al. (2023) Dupilumab increases aspirin tolerance in NSAID-exacerbated respiratory disease. The European respiratory journal 61 10.1183/13993003.01335-2022 PMC10017890 36549708

[92] Segboer CL, Fokkens WJ, Terreehorst I et al (2018) Endotyping of non-allergic, allergic and mixed rhinitis patients using a broad panel of biomarkers in nasal secretions. PloS one 13:e0200366 10.1371/journal.pone.0200366 PMC6061980 30048449

[93] Shamizadeh S, Brockow K, Ring J (2014) Rupatadine: efficacy and safety of a non-sedating antihistamine with PAF-antagonist effects. Allergo journal international 23:87–95 10.1007/s40629-014-0011-7 PMC4479428 26120520

[94] Simon Ra AN, Feldweg Am (2022) Aspirin-exacerbated respiratory disease: NSAID challenge and densensitization. UpToDate (2022). . In:UpToDate.

[95] Staudacher AG, Peters AT, Kato A et al (2020) Use of endotypes, phenotypes, and inflammatory markers to guide treatment decisions in chronic rhinosinusitis. Ann Allergy Asthma Immunol 124:318–325 10.1016/j.anai.2020.01.013 PMC7192133 32007571

[96] Staufenberg AR, Frankenberger HK, Förster-Ruhrmann U et al (2024) Biologikatherapie bei schwer erkrankten Patienten mit „NSAID-exacerbated respiratory disease“ und stattgehabter ASS-Desaktivierung : Ergebnisse einer multizentrischen Studie. Hno 72:473–483 10.1007/s00106-024-01433-y PMC11192825 38466409

[97] Stevens WW, Jerschow E, Baptist AP et al (2020) The role of aspirin desensitization followed by oral aspirin therapy in managing patients with aspirin-exacerbated respiratory disease: A Work Group Report from the Rhinitis, Rhinosinusitis and Ocular Allergy Committee of the American Academy of Allergy, Asthma & Immunology. The Journal of allergy and clinical immunology 147:827–844 10.1016/j.jaci.2020.10.043 PMC7980229 33307116

[98] Stevens WW, Peters AT, Tan BK et al (2019) Associations Between Inflammatory Endotypes and Clinical Presentations in Chronic Rhinosinusitis. The journal of allergy and clinical immunology In practice 7:2812–2820 10.1016/j.jaip.2019.05.009 PMC6842686 31128376

[99] Stevenson DD, Hankammer MA, Mathison DA et al (1996) Aspirin desensitization treatment of aspirin-sensitive patients with rhinosinusitis-asthma: long-term outcomes. The Journal of allergy and clinical immunology 98:751–758 10.1016/s0091-6749(96)70123-9 8876550

[100] Stevenson DD, Simon RA, Mathison DA (1980) Aspirin-sensitive asthma: tolerance to aspirin after positive oral aspirin challenges. The Journal of allergy and clinical immunology 66:82–88 10.1016/0091-6749(80)90143-8 7381126

[101] Sweet JM, Stevenson DD, Simon RA et al (1990) Long-term effects of aspirin desensitization--treatment for aspirin-sensitive rhinosinusitis-asthma. The Journal of allergy and clinical immunology 85:59–65 10.1016/0091-6749(90)90222-p 2299107

[102] Sweis AM, Locke TB, Ig-Izevbekhai KI et al (2020) Major complications of aspirin desensitization and maintenance therapy in aspirin-exacerbated respiratory disease. International forum of allergy & rhinology 11:115–119 10.1002/alr.22643 32671928

[103] Szczeklik A, Gryglewski RJ, Czerniawska-Mysik G (1977) Clinical patterns of hypersensitivity to nonsteroidal anti-inflammatory drugs and their pathogenesis. The Journal of allergy and clinical immunology 60:276–284 10.1016/0091-6749(77)90106-3 410857

[104] Szczeklik A, Gryglewski RJ, Czerniawska-Mysik G (1975) Relationship of inhibition of prostaglandin biosynthesis by analgesics to asthma attacks in aspirin-sensitive patients. British medical journal 1:67–69 10.1136/bmj.1.5949.67 PMC1672199 1109660

[105] Szczeklik A, Nizankowska E, Duplaga M, AIANE Investigators, European Network on Aspirin-Induced Asthma (2000) Natural history of aspirin-induced asthma. The European respiratory journal 16:432–436 10.1034/j.1399-3003.2000.016003432.x 11028656

[106] Szczeklik A, Sanak M, Nizankowska-Mogilnicka E et al (2004) Aspirin intolerance and the cyclooxygenase-leukotriene pathways. Current opinion in pulmonary medicine 10:51–56 10.1097/00063198-200401000-00009 14749606

[107] Ta V, White AA (2015) Survey-Defined Patient Experiences With Aspirin-Exacerbated Respiratory Disease. The journal of allergy and clinical immunology. In practice 3:711–718 10.1016/j.jaip.2015.03.001 25858054

[108] Taniguchi M, Mitsui C, Hayashi H et al (2019) Aspirin-exacerbated respiratory disease (AERD): Current understanding of AERD. Allergol Int 68:289–295 10.1016/j.alit.2019.05.001 31235242

[109] Taube C (2014) Bronchial asthma: is personalized therapy on the horizon? Allergo journal international 23:246–251 10.1007/s40629-014-0028-y PMC4479476 26120534

[110] Tomassen P, Vandeplas G, Van Zele T et al (2016) Inflammatory endotypes of chronic rhinosinusitis based on cluster analysis of biomarkers. The Journal of allergy and clinical immunology 137:1449–1456.e1444 10.1016/j.jaci.2015.12.1324 26949058

[111] Uzbekov R, Bouakaz A, Postema M (2023) Closeups of a not-so-domestic mite tritonymph. Allergo Journal International 32:337–339

[112] Vashishta R, Soler ZM, Nguyen SA et al (2013) A systematic review and meta-analysis of asthma outcomes following endoscopic sinus surgery for chronic rhinosinusitis. Int Forum Allergy Rhinol 3:788–794 10.1002/alr.21182 23818462

[113] Vrtala S (2022) Allergens from house dust and storage mites. Allergo Journal International 31:267–271

[114] White AA, Stevenson DD (2018) Aspirin-Exacerbated Respiratory Disease. The New England journal of medicine 379:1060–1070 10.1056/NEJMra1712125 30207919

[115] White AA, Woessner K, Simon R (2020) Aspirin-exacerbated respiratory disease: Update on medical management. World journal of otorhinolaryngology - head and neck surgery 6:241–247 10.1016/j.wjorl.2020.07.009 PMC7729248 33336180

[116] Yazici D, Ogulur I, Kucukkase O et al (2022) Epithelial barrier hypothesis and the development of allergic and autoimmune diseases. Allergo Journal International 31:91–102

[117] Zeitz HJ (1988) Bronchial asthma, nasal polyps, and aspirin sensitivity: Samter’s syndrome. Clinics in chest medicine 9:567–576 3069289

